# Design, Synthesis, Molecular Docking Analysis, and Carbonic Anhydrase IX Inhibitory Evaluations of Novel *N*-Substituted-*β*-d-Glucosamine Derivatives that Incorporate Benzenesulfonamides

**DOI:** 10.3390/molecules22050785

**Published:** 2017-05-12

**Authors:** Feng-Ran Li, Zhan-Fang Fan, Su-Jiao Qi, Yan-Shi Wang, Jian Wang, Yang Liu, Mao-Sheng Cheng

**Affiliations:** Key Laboratory of Structure-Based Drugs Design and Discovery (Ministry of Education), School of Pharmaceutical Engineering, Shenyang Pharmaceutical University, Shenyang 110016, China; lifengran_1989@163.com (F.-R.L.); FZF11201604@163.com (Z.-F.F.); qisj_90@163.com (S.-J.Q.); wangyanshispu@163.com (Y.-S.W.); jianwang@syphu.edu.cn (J.W.)

**Keywords:** *N*-substituted-*β*-d-glucosamine derivatives, benzenesulfonamides, human carbonic anhydrase IX, molecular docking

## Abstract

A series of novel *N*-substituted-*β*-d-glucosamine derivatives that incorporate benzenesulfonamides were designed using a fragment-based drug design strategy. Each derivative was synthesized and evaluated in vitro for its inhibitory activity against human carbonic anhydrase (hCA) IX; several derivatives displayed desirable potency profiles against this enzyme. The molecular docking studies provided the design rationale and predicted potential binding modes for carbonic anhydrase (CA) IX and three target compounds, including the most potent inhibitor, compound **7f** (IC_50_ = 10.01 nM). Moreover, the calculated Log P (cLog P) values showed that all the compounds tended to be hydrophilic. In addition, topological polar surface area (TPSA) value-based predictions highlighted the selectivity of these carbohydrate-based inhibitors for membrane-associated CA IX.

## 1. Introduction

Carbonic anhydrases (CAs) are ubiquitous zinc metalloenzymes that catalyze the reversible hydration of carbon dioxide and water to a bicarbonate ion and proton [1,2,3,4,5,6]. To date, human cells express twelve catalytically active CA isoforms belonging to the α family. These isoforms have been classified into four different subclasses based on the subcellular localization: cytosolic isoforms (CA I, CA II, CA III, CA VII, and CA XIII) and membrane-bound (CA IV, CA IX, CA XII, and CA XIV), secreted (CA VI), and mitochondrial (CA VA and CA VB) forms [1,2,7]. Apart from this difference, they also have variable organ and tissue distributions and catalytic activities. The members of human carbonic anhydrase (hCA) family share 30–40% sequence identity and display only minor differences in the region comprising residues 125–137 [1]. In all isoenzymes, the active site is a conical cavity consisting of two conserved regions (hydrophobic and hydrophilic) and a catalytic Zn^2+^ ion at the bottom which exhibits coordination with three His residues and the substrate H_2_O/hydroxide. The role of the zinc cation is to bind to and activate the substrate H_2_O in the process of hydration of CO_2_ [1,2].

As a hypoxia-induced transmembrane enzyme with an extracellular catalytic domain, CA IX is a tumor-associated isozyme which is upregulated in varieties of hypoxic tumor cells, while restrictedly expressed in the normal tissues [1,7]. The activity of this isozyme contributes to maintaining a neutral intracellular pH and the extracellular acidication of the solid tumor. This process favors tumor growth, metastasis, and invasion and provides a suitable environment for hypoxic tumor cells survival and proliferation [1,2,3,8,9]. Accordingly, this enzyme has been validated as a promising target for cancer diagnostics and treatments.

**Figure 1:**
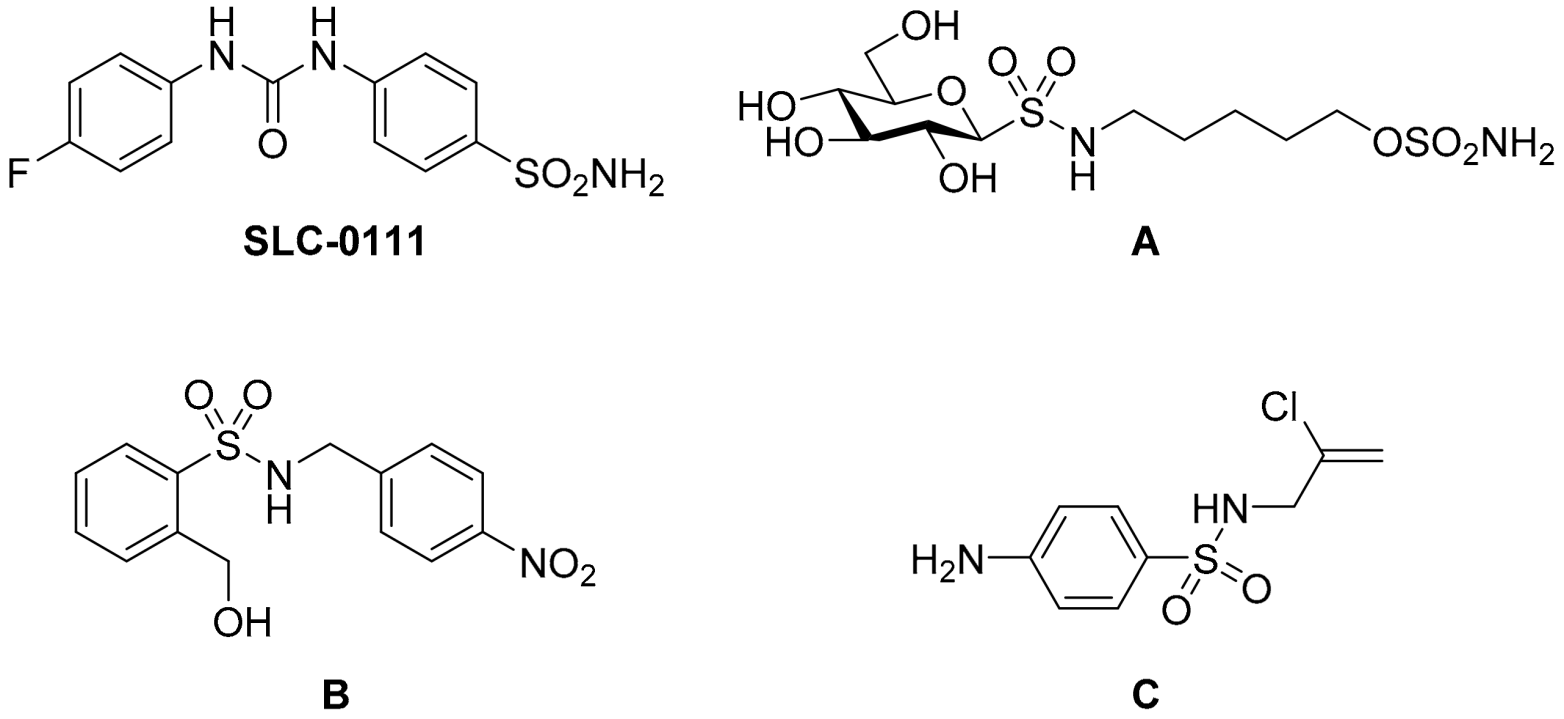
Chemical structures of SLC0111, glycoconjugate **A**, and secondary sulfonamides **B** and **C**.

**Figure 2:**
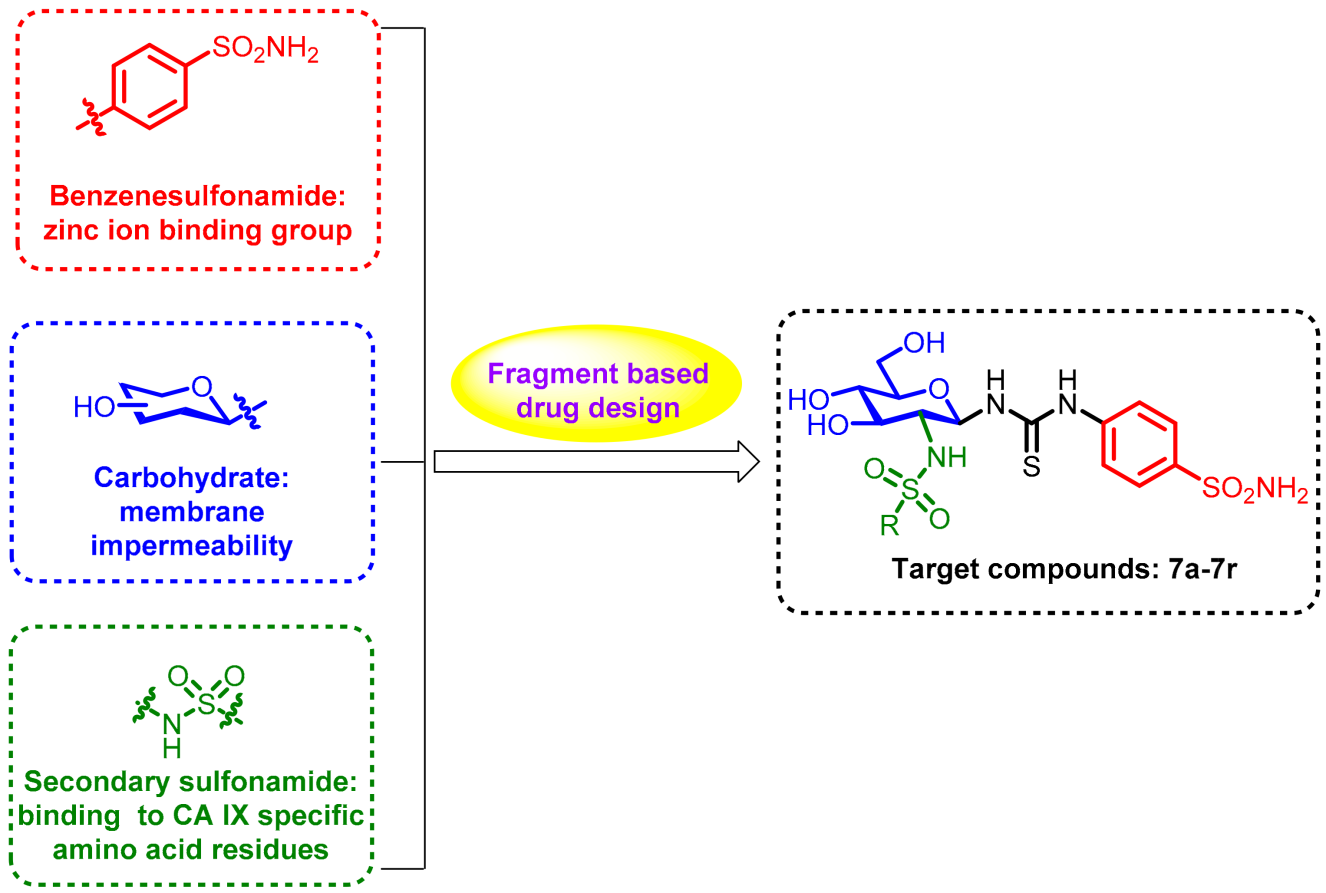
The design strategy of the target compounds.

Recently, several CA IX inhibitors have been reported. SLC0111 is a potent CA IX inhibitor and progressed into phase I clinical trials as an antitumor agent (Figure 1) [10]. Like this CA IX candidate, most inhibitors contain the classical zinc-binding-group sulfonamide (RSO_2_NH_2_) with excellent CA IX inhibition abilities; the deprotonated sulfonamide (RSO_2_NH^−^) coordinates to the active site zinc cation and so blocks the activity of the enzyme. However, owing to the high structural similarity of the active site of CA isoforms, a number of inhibitors lack selectivity for CA IX [11,12,13,14]. Supuran’s group has demonstrated the success of appending carbohydrate scaffolds to aromatic sulfonamides in the design of selective CA inhibitors, particularly for CA IX [7,15,16,17,18,19,20]. The poor membrane permeability and wide structural diversity of these glycol-inhibitors facilitates their preferential inhibition of transmembrane CA IX. Compound **A**, an exemplary glycoconjugate in this aspect, exhibits 1200-fold and 3200-fold selectivity for CA IX over intracellular CA I and CA II, respectively (Figure 1). The attachment of sugar hydrophilic moiety provides the opportunities to specifically target CA IX due to the membrane impermeability of the inhibitor [20]. In addition, secondary sulfonamide moiety has been used in numerous clinical drugs [21], and it has also been described in the literature as a potent and specific inhibitor for CA IX [12,22,23,24,25]. For example, compounds **B** and **C**, incorporating a secondary sulfonamide rather than a primary one, showed low Ki values and excellent selectivity to CA IX (Figure 1) [24,25]. Inspired by the activities of the aforementioned moieties, we used a fragment-based drug design strategy to combine aromatic sulfonamide and glucosamine with a thiourea linker. The substituted sulfonyl was introduced onto the 2-amino group of the glucosamine to simultaneously form the secondary sulfonamide group for the design of novel CA IX selective inhibitors (Figure 2). The primary sulfonamide may anchor the Zn^2+^ ion and the carbohydrate is able to decrease the membrane permeability of the whole molecule. The purpose of the attachment of a secondary sulfonamide was to interact with the amino acid residues in the active site. To investigate the contributions of the secondary sulfonamide on the inhibition of CA IX, the acetyl (Ac) substituent group was also attached to the 2-amino group of the glucosamine for comparison. Molecular docking was performed to determine the binding affinities of the designed compounds for CA IX. Each compound was assessed for its ability to inhibit human CA IX in vitro. The IC_50_ values showed that substituted sulfonyl groups possessed better activities than the acetyl substituent group. Furthermore, the hydrophilic properties and membrane permeabilities of these derivatives were predicted by the calculated Log P (cLog P) and topological polar surface area (TPSA) values, respectively.

## 2. Results and Discussion

### 2.1. Molecular Docking Studies

To elucidate the binding patterns of the designed compounds, we predicted the binding affinities by molecular docking. Compounds **7a**–**7r** and **9a**–**9b** were docked in the CA IX (Protein Data Bank (PDB): 5FL4) binding site using the Autodock 4.0 package. The clinically used drug and broad CA inhibitor, Acetazolamide (AZA), was also included as the reference due to its available co-crystal structure with CA IX and known inhibition on the enzyme (Ki = 25 nM). The binding energies were calculated using the Autodock and X-score programs and are presented in Table 1. The docking results indicated that all designed compounds exhibited lower binding energy values and higher affinities for CA IX than did AZA, suggesting that these compounds probably achieved higher or comparable activities against CA IX.

### 2.2. Predictions by cLog P and TPSA

The poor membrane permeability of each inhibitor should improve selectivity for the extracellular catalytic domain of isozyme CA IX. For our target compounds, due to the unmarked sugar moiety which contains several hydroxyl groups, we predicted an increase in the molecular polarity and a decrease the membrane permeability. Lipophilicity is an important property in drug discovery, and it is estimated by the value of Log P. The calculated Log P (cLog P) value for each compound was predicted using Discovery Studio 3.0. As shown in Table 1, the values of cLog P for all compounds, ranging from −3.6589 to 1.0453, comply to the rule of five [26] and tend to present the hydrophilic properties. Topological polar surface area (TPSA) is the calculated value of characterizing membrane permeability. A TPSA value >140 Å^2^ indicates that a molecule has poor membrane permeability [27]. A TPSA value for each compound was calculated using Discovery Studio 3.0 and the results are summarized in Table 1. Values of all derivatives are greater than 140 Å^2^ (ranging from 188.25 to 248.37). The results indicate that it is possible for the designed compounds to exhibit hydrophilic properties, limited membrane permeabilities, and specifically target CA IX.

### 2.3. Chemistry

**Scheme 1:**
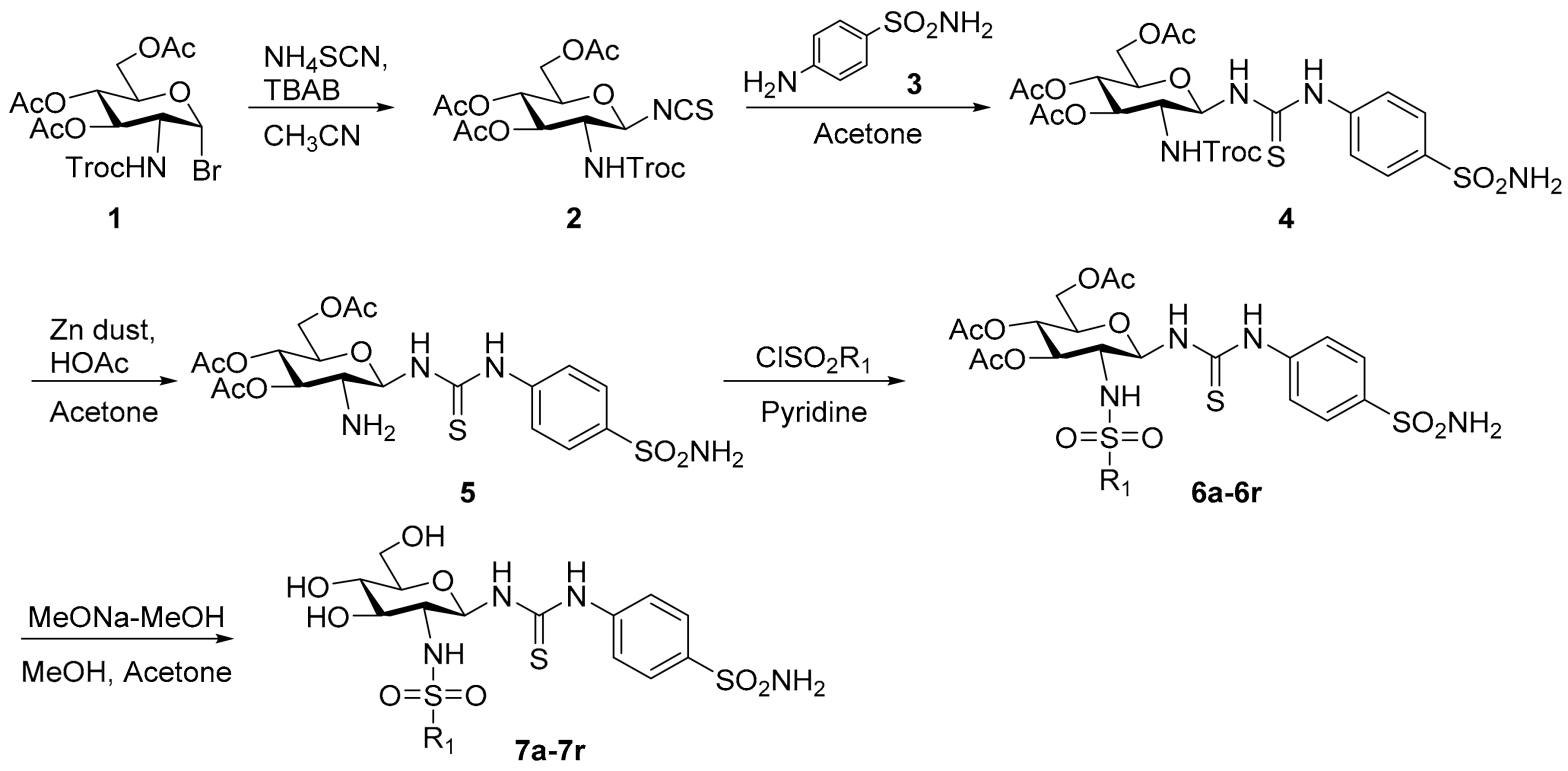
The synthesis of target compounds **7a**–**7r**.

Target compounds **7a**–**7r** were synthesized as outlined in Scheme 1. The synthetic route began with glycosyl bromide **1**, which was prepared as previously described [28]. Treatment of **1** with ammonium thiocyanate yielded isothiocyanate **2 [29]**, which was coupled with sulfonamide **3** to give thiourea compound **4**. The trichloroethoxycarbonyl moiety of compound **4** was removed with zinc powder in acetic acid [30,31]. Then, the amine **5** was condensed with substituted sulfonyl chloride to generate intermediates **6a**–**6r**, followed by deacetylation with 0.1 M sodium methoxide to generate compounds **7a**–**7r**.

**Scheme 2:**
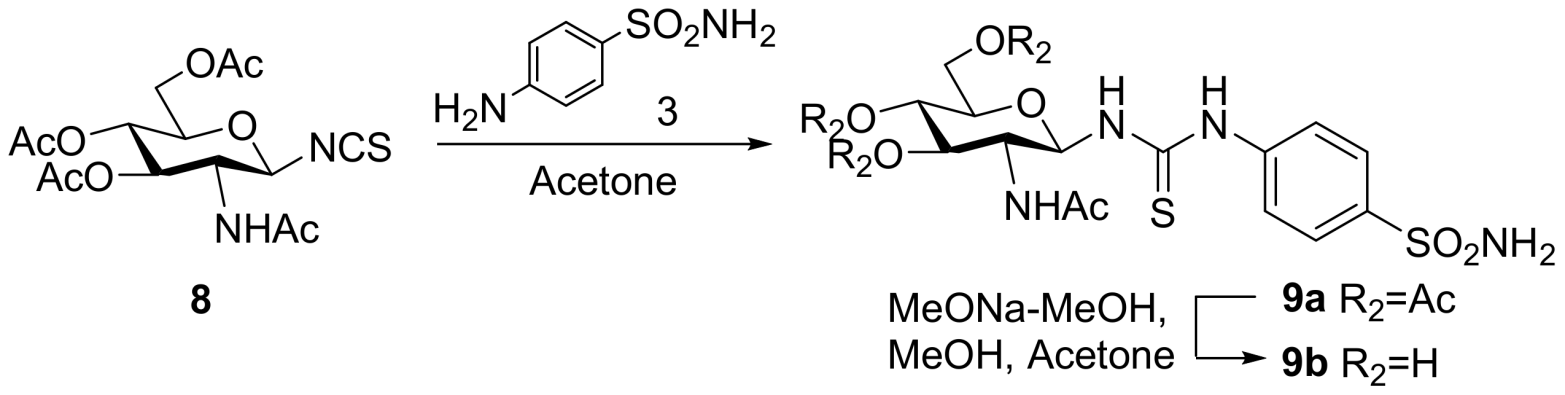
The synthesis of target compounds **9a** and **9b**.

The synthesis of compounds **9a** and **9b** followed an analogous strategy to that of compound **4** and the process is shown in Scheme 2. Isothiocyanate **8** was synthesized from a reaction between glycosyl bromide and potassium thiocyanate as previously described [32]. It was converted to thiourea compound **9a** using the aforementioned method. The hydroxyl-protecting groups were subsequently cleaved to yield **9b**.

### 2.4. Human Carbonic Anhydrase IX Inhibition Studies

**Figure 3:**
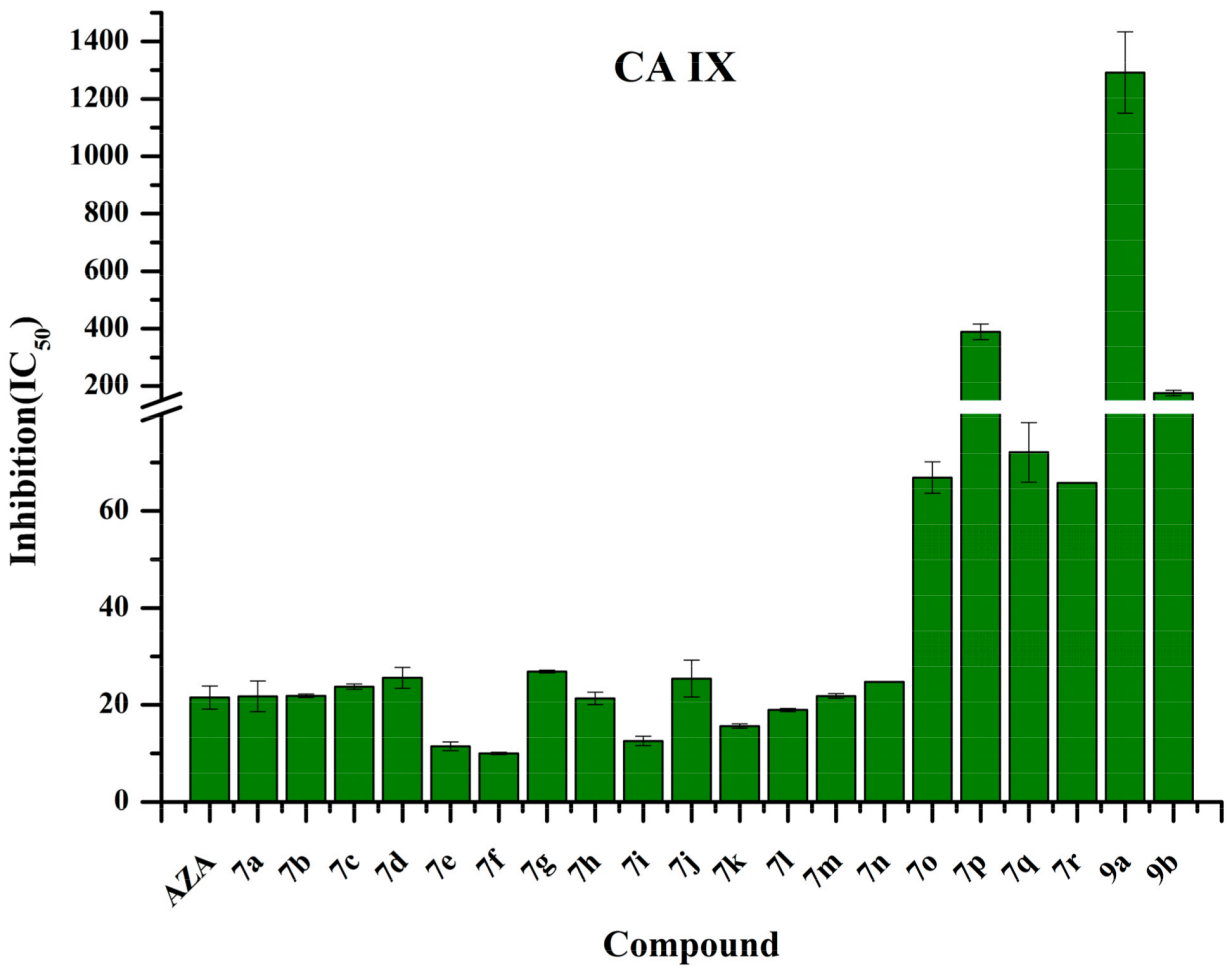
IC_50_ values of compounds **7a**–**7r** and **9a**–**9b** against carbonic anhydrase (CA) IX.

Compounds **7a**–**7r** and **9a** and **9b** were measured for their inhibition of CA IX. AZA was included in the assay as the standard inhibitor. The inhibition data are listed in Table 1 and the results are presented in Figure 3.

The IC_50_ values revealed that several conjugates exhibited higher activities against CA IX than AZA. The best CA IX inhibitor was 4-trifluoromethylbenzenesulfonyl amino derivative **7f** (IC_50_ of 10.01 nM); several other compounds (**7e**, **7i**, **7k** and **7l**) had IC_50_ values that were <20 nM, which made them effective CA IX inhibitors. The structure activity relationships (SARs) were determined as follows:

(i) The deprotected sugar analogs (**7a**–**7r** and **9a**) had IC_50_ values within 10.01–388.80 nM. Most of the compounds that contained sulfonyl substituents on the 2-amino group of their glucosamines showed stronger inhibitory activities against CA IX (up to 17.6-fold) than that of compound **9a**, which contained an acetyl group at the same position; *n*-propylsulfonyl derivative **7p** (IC_50_ of 388.80 nM) was the exception.

(ii) Regarding substitutions of the sulfonyl moiety, the phenyl and naphthyl groups exhibited higher affinities for CA IX compared to that of the other groups. However, the effects of the nature and position of the groups that were used to substitute the aromatic moiety in compounds **7a**–**7n** on CA IX inhibition were not apparent.

(iii) The acetyl-protected sugar **9a** was a weaker inhibitor than free sugar **9b**. This is closely related to its better membrane permeability.

**Figure 4:**
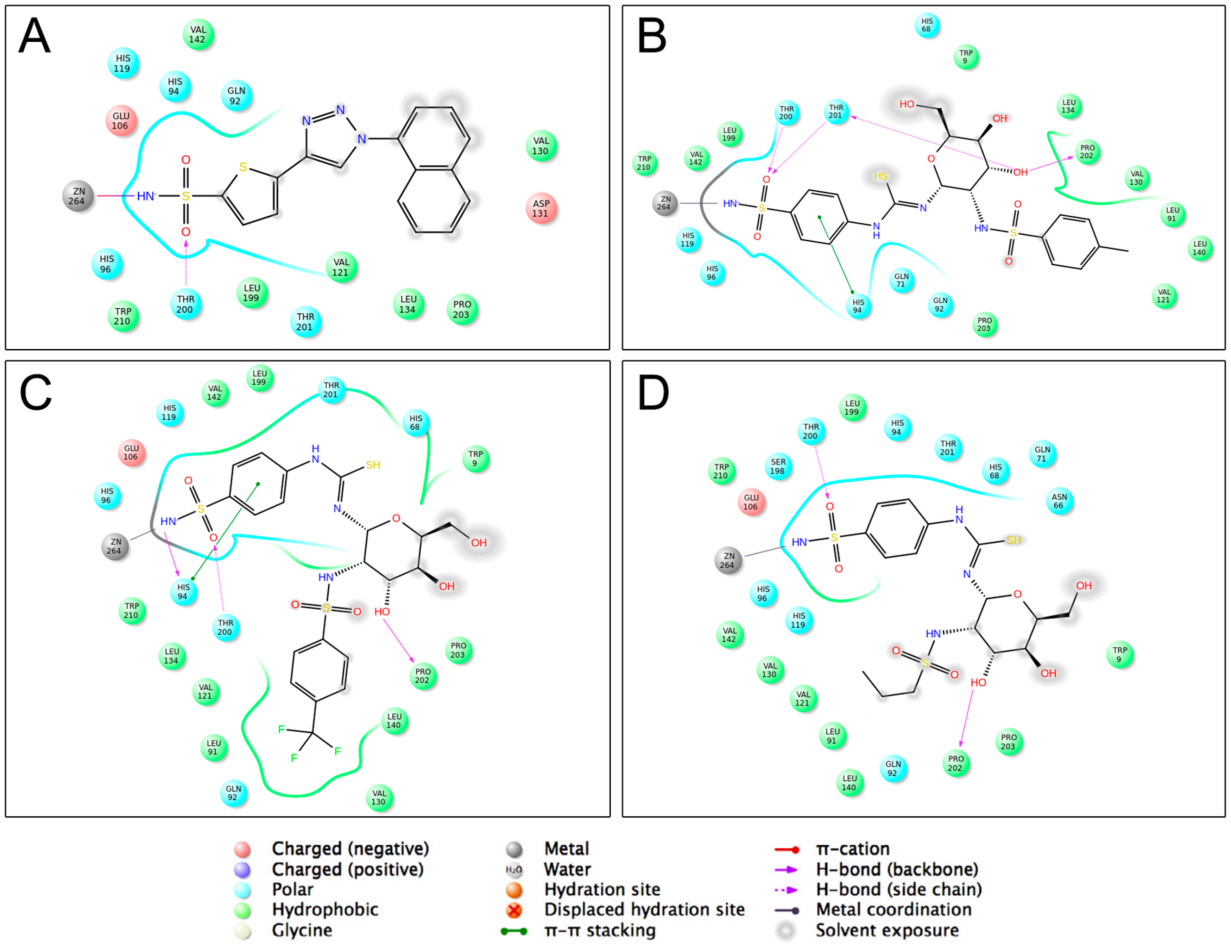
Two-dimensional docking poses of compound 5FL4-ref (**A**); compound **7a** (**B**); compound **7f** (**C**) and compound **7p** (**D**) with the active site of CA IX. The key hydrogen bonds were indicated with purple lines. The light gray sphere represented the zinc ion. The hydrophobic interactions were displayed by green lines and spheres.

Compounds **7a**, **7f**, and **7p** were chosen for the analyses of molecular interactions and the co-crystallized compound 5FL4-ref was included as a comparison. As shown in Figure 4, compound 5FL4-ref interacted with the active site of CA IX by one hydrogen bond between the Thr200 and SO_2_ group, and the nitrogen atom of the sulfonamide group formed a coordination bond with the zinc ion. These interactions are in a similar manner to the reported co-crystal structure between AZA and CA IX [1]. The naphthyl moiety of 5FL4-ref formed weak hydrophobic interactions with Val121, Val130, Leu134, and Pro203. Compound **7a**, **7f**, and **7p** were also capable of forming interactions with the zinc cation, and coordinating by means of the nitrogen atom of sulfonamide. It was also observed that the SO_2_ group and 3-hydroxyl moiety on the sugar of compounds **7a**, **7f**, and **7p** formed hydrogen bonds with the Thr200 and Pro202 residues, respectively. Regarding the aromatic substituted compounds **7a** and **7f**, the primary sulfonamide also displayed hydrogen bond interactions with other amino acid residues and the benzene ring tethering primary sulfonamide showed π-π stacking with His94, whereas compound **7p** lacked these interactions. Furthermore, the substitution on the secondary sulfonamide were positioned to the hydrophobic pocket (Leu91, Val121, Val131, Leu135, Leu141, Val143, Leu198, and Pro202) [1]. Compound **7a** and **7f** both formed stronger hydrophobic interactions with the hydrophobic pocket than did *n*-propylsulfonyl derivative **7p**. And the binding modes revealed that the aromatic ring substitutions were unable to affect the inhibition on CA IX significantly (Figure 4B,C).

## 3. Materials and Methods

### 3.1. Molecular Docking and Predictions by cLog P and TPSA

AutoDock 4 [33] was used to perform docking calculations. The crystal structures of human carbonic anhydrase IX (PDB code: 5FL4) [34] were used in docking calculations. A grid box with a grid spacing of 0.375 Å was generated to define the binding pocket. Affinity grid fields were generated using the auxiliary program AutoGrid 4. Compound structures were built and minimized with the Accelrys Discovery Studio 3.0 software package [35] with flexible torsions assigned, and all dihedral angles were allowed to freely rotate. The Lamarckian genetic algorithm was used to determine the appropriate binding positions, orientations, and conformations of ligands. The optimized parameters were as follows: the maximum number of energy evaluations was increased to 25,000,000 per run, the iterations of Solis & Wets local search were 3000, the number of individuals in the population was 300, and the number of generations was 100. Results differing by <2 Å in a positional root mean square deviation were clustered together. In each group, the lowest binding energy configuration with the highest percentage frequency was selected as the group representative. All other parameters were maintained as default. To improve the accuracy of the molecular docking calculation, both AutoDock and X-score were utilized to predict the binding free energies of the compound with CA IX in this study. Finally, calculated Log P (cLog P) and TPSA of compounds were predicted by Accelrys Discovery Studio 3.0 software package [35].

### 3.2. Chemistry

All the reagents were used without further purification unless otherwise specified. Solvents were dried and redistilled prior to use according to the standard method. Analytical thin layer chromatography (TLC) was performed using silica gel HF_254_. Preparative column chromatography was performed with silica gel H. Melting points were determined on a Büchi melting point B-540 apparatus. ^1^H- and ^13^C-NMR spectra were recorded on a Bruker ARX 600 MHz or 400 MHz spectrometer using DMSO-*d*_6_ or C_5_D_5_N as solvents and TMS (tetramethylsilane) as the internal standard. High-resolution mass spectra (HR-MS) were obtained on a Bruker micrOTOF_Q spectrometer.

#### 3.2.1. *2-(2,2,2-Trichloroethoxycarbonylamino)-3,4,6-tri-O-acetyl-2-deoxy-β-d-glucopyranosyl isothiocyanate* (**2**)

Ammonium thiocyanate (2.27 g, 29.80 mmol) was treated with tetrabutylammonium bromide (1.63 g, 5.07 mmol) and molecular sieves (4 Å, 1.00 g) in dry acetonitrile (20 mL) at room temperature for 2 h. Then, the solution of glycosyl bromide **1** (16.20 g, 29.80 mmol) in dry acetonitrile (50 mL) was added dropwise. The mixture was stirred under reflux for 1 h and cooled. The solid material was filtered off and washed with acetone. The filtrate was concentrated in vacuo and the residue was purified by column chromatograph (5:1, petroleum ether–acetone) to give compound **2** (10.60 g, 68.4%) as a white solid; m.p. 138.3–140.1 °C; ^1^H-NMR (600 MHz, DMSO-*d*_6_) *δ* 10.50 (s, 1H), 5.71 (d, *J* = 8.3 Hz, 1H), 5.48 (t, *J* = 2.6 Hz, 1H), 5.08 (d, *J* = 12.2 Hz, 1H), 5.00 (d, *J* = 12.2 Hz, 1H), 4.88 (dt, 1H), 4.67 (dq, *J* = 8.1, 3.0, 1.5 Hz, 1H), 4.17–4.10 (m, 2H), 3.69–3.64 (m, 1H), 2.08 (s, 3H), 2.02 (s, 3H), 1.99 (s, 3H); ^13^C-NMR (100 MHz, DMSO-*d*_6_) *δ* 179.73, 170.05, 169.05, 168.65, 149.33, 94.60, 79.54, 74.94, 67.66, 66.45, 66.32, 63.66, 56.23, 20.61, 20.56, 20.53; HRMS (ESI): Calcd. for [M − H]^−^ C_16_H_18_Cl_3_N_2_O_9_S_1_: 518.9799, Found 518.9794.

#### 3.2.2. *N-[4-(Aminosulfonyl)phenyl]-N′-[2′-(2′,2′,2′-trichloroethoxycarbonylamino)-3′,4′,6′-tri-O-acetyl-2′-deoxy-β-d-glucopyranosyl]thiourea* (**4**)

To a solution of the isothiocyanate intermediate **2** (5.00 g, 9.58 mmol) in dry acetonitrile (30 mL), sulfonamide **3** (3.30 g, 19.16 mmol) was added and stirred at 50 °C for 4 h. The mixture was cooled to room temperature, concentrated, and diluted with ethyl acetate. The organic layer was washed with aqueous hydrochloric acid (1N), saturated aqueous sodium bicarbonate and brine, dried over anhydrous sodium sulfate, filtered, concentrated, and purified by column chromatograph (100:1, dichloromethane–methanol) to afford compound **4** (6.20 g, 93.23%) as a white solid; m.p. 159.2–161.1 °C; ^1^H-NMR (600 MHz, DMSO-*d*_6_) *δ* 10.23 (s, 1H), 8.35 (s, 1H), 8.11 (d, *J* = 9.4 Hz, 1H), 7.75 (d, *J* = 8.6 Hz, 2H), 7.68 (d, *J* = 7.8 Hz, 2H), 7.30 (s, 2H), 5.72 (s, 1H), 5.21 (t, *J* = 9.8 Hz, 1H), 4.90–4.83 (m, 3H), 4.21 (dd, *J* = 12.4, 4.6 Hz, 1H), 3.97 (d, *J* = 12.0 Hz, 1H), 3.82 (q, *J* = 9.8 Hz, 1H), 3.76 (s, 1H), 1.99 (s, 3H), 1.98 (s, 3H), 1.92 (s, 3H); ^13^C-NMR (100 MHz, DMSO-*d*_6_) *δ* 181.86, 170.03, 169.39, 154.62, 141.92, 139.37, 126.24, 122.13, 96.21, 73.30, 73.09, 72.20, 68.56, 61.74, 54.20, 20.56, 20.43, 20.41; HRMS (ESI): Calcd. for [M + H]^+^ C_22_H_28_Cl_3_N_4_O_11_S_2_: 693.0262, Found 693.0256.

#### 3.2.3. General Procedure for the Synthesis of Compounds **7a**–**7r**

To a mixture of **4** (500 mg, 0.72 mmol) in acetone (10 mL), freshly activated zinc powder (1.65 g, 25.22 mmol) and acetic acid (10 mL) were added and stirred for 1 h. The residue was filtered, concentrated, and diluted with ethyl acetate. The organic layer was washed by saturated aqueous sodium bicarbonate and brine, dried over anhydrous sodium sulfate, filtered, and concentrated in vacuo. The white solid product **5** was directly used for the next step without further purification.

To a solution of crude **5** from the last step in dry pyridine (10 mL) at 0 °C, substituted sulfonyl chloride (0.79 mmol) was dropped into the mixture slowly, and the reaction was stirred for 10 min at room temperature. After complete consumption of the starting material, the residue was diluted with ethyl acetate and washed with aqueous hydrochloric acid (1M), saturated aqueous sodium bicarbonate, and brine. The solution was dried over anhydrous sodium sulfate, filtered, and concentrated. The light yellow oily product **6a**–**6r** was directly used for the next step without further purification.

To a solution of intermediate **6a**–**6r** in acetone–methanol (5 mL:5 mL), freshly prepared sodium methoxide in methanol solution (1.0 mol/L, 1 mL) was added. The mixture was stirred for 30 min followed by the addition of Dowex H^+^ resin to pH 7, then filtered. The filtrate was concentrated and purified by column chromatograph (10:1, dichloromethane–methanol) to obtain **7a**–**7r**.

*N-[4-(Aminosulfonyl)phenyl]-N′-[2′-(4′-methylbenzenesulfonylamino)-2′-deoxy-β-d-glucopyranosyl]thiourea* (**7a**): white solid; yield: 30.71%; m.p. 159.5–161.2 °C; ^1^H-NMR (600 MHz, DMSO-*d*_6_) *δ* 10.07 (s, 1H), 7.81 (s, 1H), 7.78–7.64 (m, 8H), 7.28 (s, 1H), 7.20 (s, 2H), 5.41 (s, 1H), 4.96 (s, 1H), 4.80 (s, 1H), 4.51 (s, 1H), 3.61 (d, *J* = 11.5 Hz, 1H), 3.44 (dd, *J* = 11.8, 4.5 Hz, 1H), 3.32–3.29 (m, 1H), 3.13–3.08 (m, 3H), 2.17 (s, 3H); ^13^C-NMR (150 MHz, DMSO-*d*_6_) *δ* 181.30, 142.33, 141.53, 140.13, 138.89, 128.91, 126.28, 126.09, 121.88, 82.70, 78.38, 74.94, 70.31, 60.50, 58.90, 20.83; HRMS (ESI): Calcd. for [M + Na]^+^ C_20_H_26_N_4_Na_1_O_8_S_3_: 569.0811, Found 569.0805.

*N-[4-(Aminosulfonyl)phenyl]-N′-[2′-(2′-methylbenzenesulfonylamino)-2′-deoxy-β-d-glucopyranosyl]thiourea* (**7b**): white solid; yield: 31.98%; m.p. 175.2–177.1 °C; ^1^H-NMR (600 MHz, DMSO-*d*_6_) *δ* 9.95 (s, 1H), 7.95 (s, 1H), 7.89 (d, *J* = 7.7 Hz, 1H), 7.76 (d, *J* = 8.7 Hz, 2H), 7.74–7.72 (m, 1H), 7.69 (d, *J* = 8.8 Hz, 2H), 7.29 (s, 2H), 7.28–7.26 (m, 1H), 7.23 (d, *J* = 3.4 Hz, 2H), 5.47 (s, 1H), 4.96 (s, 1H), 4.79 (d, *J* = 6.0 Hz, 1H), 4.50 (s, 1H), 3.60 (dd, *J* = 11.3, 4.8 Hz, 1H), 3.48–3.41 (m, 1H), 3.40–3.36 (m, 1H), 3.09–3.05 (m, 3H), 2.58 (s, 3H); ^13^C NMR (150 MHz, DMSO-*d*_6_) *δ* 181.41, 142.14, 139.05, 136.56, 136.51, 131.87, 131.54, 127.81, 126.11, 125.53, 122.05, 82.61, 78.37, 74.78, 70.41, 60.45, 58.64, 20.09; HRMS (ESI): Calcd. for [M + Na]^+^ C_20_H_26_N_4_Na_1_O_8_S_3_: 569.0811, Found 569.0805.

*N-[4-(Aminosulfonyl)phenyl]-N′-[2′-(4′-methoxybenzenesulfonylamino)-2′-deoxy-β-d-glucopyranosyl]thiourea* (**7c**): white solid; yield: 32.84%; m.p. 165.0–166.3 °C; ^1^H-NMR (600 MHz, DMSO-*d*_6_) *δ* 10.13 (s, 1H), 7.84 (s, 1H), 7.78–7.70 (m, 7H), 7.29 (s, 2H), 6.92 (d, *J* = 6.5 Hz, 2H), 5.41 (s, 1H), 4.96 (s, 1H), 4.78 (d, *J* = 2.7 Hz, 1H), 4.51 (s, 1H), 3.65 (s, 3H), 3.62–3.58 (m, 1H), 3.47–3.41 (m, 1H), 3.30–3.26 (m, 1H), 3.16–3.03 (m, 3H); ^13^C NMR (150 MHz, DMSO-*d*_6_) *δ* 181.37, 161.50, 142.17, 139.02, 134.63, 128.40, 126.11, 121.96, 113.56, 82.70, 78.37, 74.84, 70.28, 60.45, 58.76, 55.30; HRMS (ESI): Calcd. for [M + Na]^+^ C_20_H_26_N_4_Na_1_O_9_S_3_: 585.0760, Found 585.0754.

*N-[4-(Aminosulfonyl)phenyl]-N′-[2′-(4′-fluorobenzenesulfonylamino)-2′-deoxy-β-d-glucopyranosyl]thiourea* (**7d**): white solid; yield: 34.96%; m.p. 178.0–179.6 °C; ^1^H-NMR (600 MHz, DMSO-*d*_6_) *δ* 10.14 (s, 1H), 8.03 (s, 1H), 7.88–7.83 (m, 3H), 7.76 (d, *J* = 8.8 Hz, 2H), 7.72 (d, *J* = 8.8 Hz, 2H), 7.30 (s, 2H), 7.26 (t, *J* = 8.7 Hz, 2H), 5.43 (s, 1H), 4.96 (d, *J* = 3.6 Hz, 1H), 4.81 (d, *J* = 6.0 Hz, 1H), 4.51 (s, 1H), 3.60 (dd, *J* = 11.0, 3.8 Hz, 1H), 3.47–3.42 (m, 1H), 3.31–3.25 (m, 1H), 3.13–3.07 (m, 3H); ^13^C-NMR (150 MHz, DMSO-*d*_6_) *δ* 181.54, 163.64 (d, *J* = 246.0 Hz), 142.09, 139.16, 139.15 (d, *J* = 1.5 Hz), 129.22 (d, *J* = 9.0 Hz), 126.16, 122.12, 115.51 (d, *J* = 22.5 Hz), 82.69, 78.40, 74.94, 70.29, 60.44, 58.90; HRMS (ESI): Calcd. for [M + Na]^+^ C_19_H_23_F_1_N_4_Na_1_O_8_S_3_: 573.0560, Found 573.0554.

*N-[4-(Aminosulfonyl)phenyl]-N′-[2′-(3′,5′-difluorobenzenesulfonylamino)-2′-deoxy-β-d-glucopyranosyl]thiourea* (**7e**): white solid; yield: 34.47%; m.p. 164.4–165.2 °C; ^1^H-NMR (600 MHz, DMSO-*d*_6_) *δ* 10.08 (s, 1H), 8.32 (s, 1H), 7.88 (d, *J* = 9.2 Hz, 1H), 7.76 (d, *J* = 8.4 Hz, 2H), 7.71 (d, *J* = 8.8 Hz, 2H), 7.45 (d, *J* = 4.4 Hz, 2H), 7.33 (s, 1H), 7.30 (s, 2H), 5.45 (s, 1H), 5.00 (d, *J* = 5.4 Hz, 1H), 4.96 (d, *J* = 6.3 Hz, 1H), 4.52 (s, 1H), 3.61 (dd, *J* = 11.0, 4.9 Hz, 1H), 3.46–3.43 (m, 1H), 3.30–3.25 (m, 1H), 3.17–3.11 (m, 2H), 3.07 (s, 1H); ^13^C NMR (150 MHz, DMSO-*d*_6_) *δ* 181.48, 161.77 (d, *J* = 247.5 Hz), 161.69 (d, *J* = 247.5 Hz), 146.29 (d, *J* = 1.5 Hz), 142.08, 139.14, 126.19, 121.88, 109.93 (d, *J* = 28.5 Hz), 107.14 (t, *J* =25.5 Hz), 82.51, 78.48, 75.08, 70.26, 60.43, 59.17; HRMS (ESI): Calcd. for [M + Na]^+^ C_19_H_22_F_2_N_4_Na_1_O_8_S_3_: 591.0466, Found 591.0460.

*N-[4-(Aminosulfonyl)phenyl]-N′-[2′-(4′-trifluoromethylbenzenesulfonylamino)-2′-deoxy-β-d-glucopyranosyl]thiourea* (**7f**): white solid; yield: 35.88%; m.p. 164.0–165.4 °C; ^1^H-NMR (600 MHz, DMSO-*d*_6_) *δ* 10.14 (s, 1H), 8.30 (d, *J* = 8.3 Hz, 1H), 7.99 (d, *J* = 8.1 Hz, 2H), 7.90 (d, *J* = 9.0 Hz, 1H), 7.82 (d, *J* = 8.1 Hz, 2H), 7.75 (d, *J* = 8.8 Hz, 2H), 7.70 (d, *J* = 8.8 Hz, 2H), 7.30 (s, 2H), 5.45 (s, 1H), 4.96 (d, *J* = 4.9 Hz, 1H), 4.83 (d, *J* = 6.1 Hz, 1H), 4.52 (s, 1H), 3.60 (dd, *J* = 11.1, 4.7 Hz, 1H), 3.47–3.42 (m, 1H), 3.30–3.25 (m, 1H), 3.19–3.03 (m, 3H); ^13^C-NMR (150 MHz, DMSO-*d*_6_) *δ* 181.59, 146.60, 142.02, 139.20, 131.32 (q, *J* = 31.5 Hz) 127.14, 126.15, 125.68 (q, *J* = 3.0 Hz), 123.62 (q, *J* = 271.5 Hz), 122.07, 82.59, 78.42, 75.04, 70.28, 60.41, 59.05; HRMS (ESI): Calcd. for [M + Na]^+^ C_20_H_23_F_3_N_4_Na_1_O_8_S_3_: 623.0528, Found 623.0522.

*N-[4-(Aminosulfonyl)phenyl]-N′-[2′-(4′-chlorobenzenesulfonylamino)-2′-deoxy-β-d-glucopyranosyl]thiourea* (**7g**): white solid; yield: 34.07%; m.p. 167.2–168.8 °C; ^1^H-NMR (600 MHz, DMSO-*d*_6_) *δ* 10.09 (s, 1H), 8.11 (s, 1H), 7.85 (d, *J* = 8.9 Hz, 1H), 7.79 (d, *J* = 8.4 Hz, 2H), 7.76 (d, *J* = 8.7 Hz, 2H), 7.70 (d, *J* = 8.8 Hz, 2H), 7.49 (d, *J* = 8.2 Hz, 2H), 7.29 (s, 2H), 5.43 (s, 1H), 4.96 (d, *J* = 4.6 Hz, 1H), 4.83 (d, *J* = 6.0 Hz, 1H), 4.51 (s, 1H), 3.60 (dd, *J* = 10.9, 4.5 Hz, 1H), 3.47–3.41 (m, 1H), 3.28–3.27 (m, 1H), 3.14–3.04 (m, 3H); ^13^C-NMR (150 MHz, DMSO-*d*_6_) *δ* 181.50, 142.06, 141.68, 139.13, 136.34, 128.57, 128.18, 126.18, 122.08, 82.62, 78.41, 74.97, 70.30, 60.44, 58.97; HRMS (ESI): Calcd. for [M + Na]^+^ C_19_H_23_Cl_1_N_4_Na_1_O_8_S_3_: 589.0264, Found 589.0259.

*N-[4-(Aminosulfonyl)phenyl]-N′-[2′-(4′-bromobenzenesulfonylamino)-2′-deoxy-β-d-glucopyranosyl]thiourea* (**7h**): white solid; yield: 33.41%; m.p. 174.1–176.0 °C; ^1^H-NMR (600 MHz, DMSO-*d*_6_) *δ* 10.08 (s, 1H), 8.11 (d, *J* = 7.4 Hz, 1H), 7.84 (d, *J* = 9.0 Hz, 1H), 7.76 (d, *J* = 8.8 Hz, 2H), 7.73–7.69 (m, 4H), 7.63 (d, *J* = 8.2 Hz, 2H), 7.29 (s, 2H), 5.43 (s, 1H), 4.96 (d, *J* = 4.9 Hz, 1H), 4.84 (d, *J* = 6.1 Hz, 1H), 4.51 (s, 1H), 3.60 (dd, *J* = 11.0, 4.8 Hz, 1H), 3.46–3.40 (m, 1H), 3.31–3.25 (m, 1H), 3.14–3.08 (m, 2H), 3.07 (s, 1H); ^13^C-NMR (150 MHz, DMSO-*d*_6_) *δ* 181.49, 142.06, 139.12, 131.50, 128.29, 126.21, 125.26, 122.08, 82.61, 78.41, 74.97, 70.30, 60.44, 58.98; HRMS (ESI): Calcd. for [M + Na]^+^ C_19_H_23_Br_1_N_4_Na_1_O_8_S_3_: 632.9759, Found 632.9754.

*N-[4-(Aminosulfonyl)phenyl]-N′-[2′-(3′-bromobenzenesulfonylamino)-2′-deoxy-β-d-glucopyranosyl]thiourea* (**7i**): white solid; yield: 34.55%; m.p. 172.9–174.7 °C; ^1^H-NMR (600 MHz, DMSO-*d*_6_) *δ* 10.05 (s, 1H), 8.17 (s, 1H), 7.95 (s, 1H), 7.86 (d, *J* = 9.1 Hz, 1H), 7.78 (d, *J* = 8.8 Hz, 1H), 7.76 (d, *J* = 8.8 Hz, 2H), 7.71 (d, *J* = 8.8 Hz, 2H), 7.61 (d, *J* = 6.3 Hz, 1H), 7.39 (t, *J* = 7.8 Hz, 1H), 7.29 (s, 2H), 5.44 (s, 1H), 4.98 (d, *J* = 5.1 Hz, 1H), 4.89 (d, *J* = 6.2 Hz, 1H), 4.51 (s, 1H), 3.60 (dd, *J* = 11.1, 4.4 Hz, 1H), 3.47–3.41 (m, 1H), 3.31–3.25 (m, 1H), 3.18–3.10 (m, 2H), 3.06 (s, 1H); ^13^C-NMR (150 MHz, DMSO-*d*_6_) *δ* 181.46, 144.81, 142.12, 139.07, 134.28, 130.69, 128.76, 126.11, 125.18, 122.03, 121.50, 82.58, 78.43, 75.03, 70.27, 60.42, 59.01; HRMS (ESI): Calcd. for [M + Na]^+^ C_19_H_23_Br_1_N_4_Na_1_O_8_S_3_: 632.9759, Found 632.9754.

*N-[4-(Aminosulfonyl)phenyl]-N′-[2′-(4′-nitrobenzenesulfonylamino)-2′-deoxy-β-d-glucopyranosyl]thiourea* (**7j**): white solid; yield: 30.77%; m.p. 174.7–176.0 °C; ^1^H-NMR (600 MHz, DMSO-*d*_6_) *δ* 10.05 (s, 1H), 8.43 (s, 1H), 8.27 (d, *J* = 8.7 Hz, 2H), 8.01 (d, *J* = 8.7 Hz, 2H), 7.92 (d, *J* = 9.0 Hz, 1H), 7.73 (d, *J* = 8.7 Hz, 2H), 7.66 (d, *J* = 8.7 Hz, 2H), 7.29 (s, 2H), 5.45 (s, 1H), 4.97 (d, *J* = 5.1 Hz, 1H), 4.86 (d, *J* = 6.0 Hz, 1H), 4.51 (s, 1H), 3.60 (dd, *J* = 11.1, 4.6 Hz, 1H), 3.46–3.41 (m, 1H), 3.29–3.25 (m, 1H), 3.15 (t, *J* = 9.6 Hz, 1H), 3.12–3.03 (m, 2H); ^13^C-NMR (150 MHz, DMSO-*d*_6_) *δ* 181.49, 148.76, 148.42, 141.93, 139.19, 127.74, 126.15, 123.89, 121.92, 82.49, 78.45, 75.06, 70.27, 60.41, 59.18; HRMS (ESI): Calcd. for [M + Na]^+^ C_19_H_23_N_5_Na_1_O_10_S_3_: 600.0505, Found 600.0499.

*N-[4-(Aminosulfonyl)phenyl]-N′-[2′-(2′-nitrobenzenesulfonylamino)-2′-deoxy-β-d-glucopyranosyl]thiourea* (**7k**): light yellow solid; yield: 32.45%; m.p. 169.7–171.7 °C; ^1^H-NMR (600 MHz, DMSO-*d*_6_) *δ* 10.15 (s, 1H), 8.26 (s, 1H), 8.12 (d, *J* = 7.7 Hz, 1H), 7.87 (d, *J* = 7.7 Hz, 1H), 7.84 (d, *J* = 9.1 Hz, 1H), 7.76 (d, *J* = 8.7 Hz, 2H), 7.74–7.65 (m, 4H), 7.29 (s, 2H), 5.57 (s, 1H), 4.97 (d, *J* = 4.1 Hz, 1H), 4.91 (d, *J* = 6.1 Hz, 1H), 4.52 (s, 1H), 3.61 (dd, *J* = 11.2, 4.8 Hz, 1H), 3.47–3.40 (m, 2H), 3.21 (t, *J* = 9.7 Hz, 1H), 3.13–3.05 (m, 2H); ^13^C-NMR (150 MHz, DMSO-*d*_6_) *δ* 181.61, 146.94, 142.11, 139.13, 134.97, 132.97, 132.32, 130.11, 126.19, 124.08, 122.09, 82.31, 78.38, 74.80, 70.40, 60.43, 59.36; HRMS (ESI): Calcd. for [M − H]^−^ C_19_H_22_N_5_O_10_S_3_: 576.0529, Found 576.0531.

*N-[4-(Aminosulfonyl)phenyl]-N′-[2′-(3′-nitrobenzenesulfonylamino)-2′-deoxy-β-d-glucopyranosyl]thiourea* (**7l**): white solid; yield: 31.25%; m.p. 177.3–179.2 °C; ^1^H-NMR (600 MHz, DMSO-*d*_6_) *δ* 10.04 (s, 1H), 8.55 (s, 1H), 8.43 (s, 1H), 8.24 (d, *J* = 7.2 Hz, 1H), 8.16 (d, *J* = 7.7 Hz, 1H), 7.93 (d, *J* = 9.1 Hz, 1H), 7.77–7.72 (m, 3H), 7.68 (d, *J* = 8.7 Hz, 2H), 7.30 (s, 2H), 5.45 (s, 1H), 4.98 (d, *J* = 5.1 Hz, 1H), 4.86 (d, *J* = 6.2 Hz, 1H), 4.51 (s, 1H), 3.60 (dd, *J* = 11.3, 4.9 Hz, 1H), 3.46–3.41 (m, 1H), 3.28–3.23 (m, 1H), 3.14 (t, *J* = 9.7 Hz, 1H), 3.11–3.03 (m, 2H); ^13^C-NMR (150 MHz, DMSO-*d*_6_) *δ* 181.45, 147.43, 144.47, 141.99, 139.13, 132.17, 130.40, 126.18, 125.99, 121.82, 121.36, 82.44, 78.43, 75.11, 70.22, 60.41, 59.18; HRMS (ESI): Calcd. for [M + Na]^+^ C_19_H_23_N_5_Na_1_O_10_S_3_: 600.0505, Found 600.0499.

*N-[4-(Aminosulfonyl)phenyl]-N′-[2′-(4′-cyanobenzenesulfonylamino)-2′-deoxy-β-d-glucopyranosyl]thiourea* (**7m**): white solid; yield: 32.17%; m.p. 169.0–170.6 °C; ^1^H-NMR (600 MHz, DMSO-*d*_6_) *δ* 10.09 (s, 1H), 8.35 (s, 1H), 7.93 (s, 4H), 7.89 (d, *J* = 9.1 Hz, 1H), 7.77 (d, *J* = 8.7 Hz, 2H), 7.69 (d, *J* = 8.7 Hz, 2H), 7.29 (s, 2H), 5.44 (s, 1H), 4.96 (d, *J* = 5.0 Hz, 1H), 4.84 (d, *J* = 6.1 Hz, 1H), 4.51 (s, 1H), 3.60 (dd, *J* = 11.0, 4.9 Hz, 1H), 3.47–3.41 (m, 1H), 3.29–3.23 (m, 1H), 3.17–3.03 (m, 3H); ^13^C-NMR (150 MHz, DMSO-*d*_6_) *δ* 181.58, 146.83, 141.98, 139.23, 132.70, 127.00, 126.23, 122.14, 117.93, 113.88, 82.56, 78.44, 75.05, 70.27, 60.41, 59.09; HRMS (ESI): Calcd. for [M + Na]^+^ C_20_H_23_N_5_Na_1_O_8_S_3_: 580.0606, Found 580.0601.

*N-[4-(Aminosulfonyl)phenyl]-N′-[2′-(naphthalene-2′-sulfonylamino)-2′-deoxy-β-d-glucopyranosyl]thiourea* (**7n**): white solid; yield: 34.76%; m.p. 182.7–184.6 °C; ^1^H-NMR (600 MHz, DMSO-*d*_6_) *δ* 9.94 (s, 1H), 8.41 (s, 1H), 8.08 (s, 1H), 8.01 (d, *J* = 7.3 Hz, 1H), 7.95 (d, *J* = 8.5 Hz, 1H), 7.88–7.81 (m, 3H), 7.66 (d, *J* = 8.0 Hz, 2H), 7.58–7.46 (m, 4H), 7.29 (s, 2H), 5.46 (s, 1H), 4.94 (d, *J* = 4.5 Hz, 1H), 4.82 (d, *J* = 5.2 Hz, 1H), 4.50 (s, 1H), 3.63–3.57 (m, 1H), 3.47–3.41 (m, 1H), 3.32–3.30 (m, 1H), 3.24–3.16 (m, 1H), 3.15–3.04 (m, 2H); ^13^C-NMR (150 MHz, DMSO-*d*_6_) *δ* 181.35, 141.95, 139.91, 138.92, 133.84, 131.49, 129.04, 128.53, 128.11, 127.62, 127.04, 126.35, 125.99, 122.66, 121.82, 82.69, 78.37, 74.93, 70.31, 60.45, 58.95; HRMS (ESI): Calcd. for [M + Na]^+^ C_23_H_26_N_4_Na_1_O_8_S_3_: 605.0811, Found 605.0805.

*N-[4-(Aminosulfonyl)phenyl]-N′-(2′-ethylsulfonylamino-2′-deoxy-β-d-glucopyranosyl)thiourea* (**7o**): white solid; yield: 43.55%; m.p. 164.8–166.2 °C; ^1^H-NMR (600 MHz, DMSO-*d*_6_) *δ* 10.38 (s, 1H), 7.87 (d, *J* = 8.2 Hz, 1H), 7.76 (d, *J* = 8.6 Hz, 2H), 7.73 (d, *J* = 8.7 Hz, 2H), 7.50 (s, 1H), 7.29 (s, 2H), 5.40 (s, 1H), 5.25 (d, *J* = 6.0 Hz, 1H), 5.06 (s, 1H), 4.53 (s, 1H), 3.62 (d, *J* = 6.3 Hz, 1H), 3.51–3.45 (m, 1H), 3.20–3.11 (m, 2H), 3.09–3.05 (m, 2H), 3.03–3.00 (m, 1H), 1.22 (t, *J* = 7.1 Hz, 3H); ^13^C-NMR (150 MHz, DMSO-*d*_6_) *δ* 181.82, 142.16, 139.27, 126.26, 122.39, 82.79, 78.34, 75.33, 70.50, 60.46, 58.53, 47.39, 8.24; HRMS (ESI): Calcd. for [M + Na]^+^ C_15_H_24_N_4_Na_1_O_8_S_3_: 507.0654, Found 507.0648.

*N-[4-(Aminosulfonyl)phenyl]-N′-(2′-n-propylsulfonylamino-2′-deoxy-β-d-glucopyranosyl)thiourea* (**7p**): white solid; yield: 38.44%; m.p. 159.1–160.6 °C; ^1^H-NMR (600 MHz, DMSO-*d*_6_) *δ* 10.48 (s, 1H), 7.96 (s, 1H), 7.75 (s, 4H), 7.50 (s, 1H), 7.30 (s, 2H), 5.41 (s, 1H), 5.26 (s, 1H), 5.08 (s, 1H), 4.55 (s, 1H), 3.62 (d, *J* = 9.6 Hz, 1H), 3.49–3.44 (m, 1H), 3.42–3.36 (m, 1H), 3.14–3.01 (m, 5H), 1.82–1.73 (m, 1H), 1.69–1.63 (m, 1H), 0.92 (t, *J* = 7.3 Hz, 3H); ^13^C-NMR (150 MHz, DMSO-*d*_6_) *δ* 181.82, 142.22, 139.17, 126.25, 122.25, 82.77, 78.36, 75.31, 70.46, 60.43, 58.54, 54.77, 17.04, 12.79; HRMS (ESI): Calcd. for [M + H]^+^ C_16_H_27_N_4_O_8_S_3_: 499.0991, Found 499.0986.

*N-[4-(Aminosulfonyl)phenyl]-N′-[2′-(N′,N′-dimethylsulfonylamino)-2′-deoxy-β-d-glucopyranosyl]thiourea* (**7q**): white solid; yield: 35.00%; m.p. 157.4–159.4 °C; ^1^H-NMR (600 MHz, DMSO-*d*_6_) *δ* 10.39 (s, 1H), 7.83 (s, 1H), 7.76 (d, *J* = 8.8 Hz, 2H), 7.73 (d, *J* = 8.8 Hz, 2H), 7.40 (s, 1H), 7.29 (s, 2H), 5.39 (s, 1H), 5.28 (s, 1H), 5.05 (s, 1H), 4.53 (s, 1H), 3.62 (d, *J* = 4.9 Hz, 1H), 3.50–3.44 (m, 1H), 3.40–3.34 (m, 1H), 3.20–3.15 (m, 1H), 3.10 (s, 1H), 3.02 (dd, *J* = 18.5, 9.4 Hz, 1H), 2.72 (s, 6H); ^13^C-NMR (150 MHz, DMSO-*d*_6_) *δ* 181.73, 142.22, 139.23, 126.19, 122.40, 83.05, 78.38, 74.81, 70.49, 60.45, 58.83, 37.78; HRMS (ESI): Calcd. for [M + Na]^+^ C_15_H_25_N_5_Na_1_O_8_S_3_: 522.0763, Found 522.0757.

*N-[4-(Aminosulfonyl)phenyl]-N′-[2′-(thiophene-2′-sulfonylamino)-2′-deoxy-β-d-glucopyranosyl]thiourea* (**7r**): white solid; yield: 31.44%; m.p. 172.8–174.0 °C; ^1^H-NMR (600 MHz, DMSO-*d*_6_) *δ* 10.23 (s, 1H), 8.15 (s, 1H), 7.86 (d, *J* = 8.8 Hz, 1H), 7.79–7.75 (m, 3H), 7.73 (d, *J* = 8.8 Hz, 2H), 7.62 (d, *J* = 2.2 Hz, 1H), 7.30 (s, 2H), 7.04 (t, *J* = 3.6 Hz, 1H), 5.45 (s, 1H), 4.98 (d, *J* = 4.1 Hz, 1H), 4.84 (d, *J* = 5.9 Hz, 1H), 4.53 (s, 1H), 3.61 (d, *J* = 6.1 Hz, 1H), 3.48–3.43 (m, 1H), 3.32–3.29 (m, 1H), 3.18–3.11 (m, 2H), 3.09 (s, 1H); ^13^C-NMR (150 MHz, DMSO-*d*_6_) *δ* 181.65, 143.49, 142.17, 139.20, 131.53, 131.32, 127.02, 126.19, 122.33, 82.72, 78.44, 74.71, 70.28, 60.43, 58.94; HRMS (ESI): Calcd. for [M + Na]^+^ C_17_H_22_N_4_Na_1_O_8_S_3_: 561.0218, Found 561.0213.

#### 3.2.4. *N-[4-(Aminosulfonyl)phenyl]-N′-(2′-acetylamino-3′,4′,6′-tri-O-acetyl-2′-deoxy-β-d-glucopyranosyl)thiourea* (**9a**)

To a solution of isothiocyanate intermediate **8** (5.00 g, 12.87 mmol) in dry acetone (15 mL), sulfonamide **3** (3.32 g, 19.31 mmol) was added. The mixture was stirred under reflux for 2 h. The solution was cooled to room temperature, concentrated, and diluted with ethyl acetate. The organic phase was washed with aqueous hydrochloric acid (1N), saturated aqueous sodium bicarbonate, and brine, dried over anhydrous sodium sulfate, filtered, concentrated, and purified by column chromatograph (100:1, dichloromethane–methanol) to give **9a** (7.03 g, 97.50%) as a white solid; m.p. 141.8–142.9 °C; ^1^H-NMR (600 MHz, DMSO-*d*_6_) *δ* 10.27 (s, 1H), 8.26 (d, *J* = 5.4 Hz, 1H), 8.16 (d, *J* = 9.3 Hz, 1H), 7.75 (d, *J* = 8.7 Hz, 2H), 7.72 (d, *J* = 8.4 Hz, 2H), 7.29 (s, 2H), 5.61 (t, *J* = 8.8 Hz, 1H), 5.15 (t, *J* = 9.8 Hz, 1H), 4.86 (t, *J* = 9.7 Hz, 1H), 4.20 (dd, *J* = 12.4, 4.6 Hz, 1H), 4.07 (d, *J* = 9.1 Hz, 1H), 3.97 (d, *J* = 11.9 Hz, 1H), 3.82 (d, *J* = 3.9 Hz, 1H), 1.99 (s, 3H), 1.98 (s, 3H), 1.94 (s, 3H), 1.81 (s, 3H); ^13^C-NMR (100 MHz, DMSO-*d*_6_) *δ* 181.88, 170.03, 169.89, 169.57, 169.38, 142.07, 139.30, 126.22, 122.09, 82.23, 73.23, 72.17, 68.57, 61.77, 51.68, 22.67, 20.56, 20.46, 20.37; HRMS (ESI): Calcd. for [M + Na]^+^ C_21_H_28_N_4_Na_1_O_10_S_2_: 583.1144, Found 583.1139.

#### 3.2.5. *N-[4-(Aminosulfonyl)phenyl]-N′-(2′-acetylamino-2′-deoxy-β-d-glucopyranosyl)thiourea* (**9b**)

To a solution of intermediate **9a** (500 mg, 0.89 mmol) in acetone-methanol (10 mL: 10 mL), freshly prepared sodium methoxide in methanol solution (1.0 mol/L, 2 mL) was added. After it was stirred for 30 min, the mixture was neutralized with Dowex H^+^ resin to pH 7, then filtered. The filtrate was concentrated and purified by column chromatograph (10:1, dichloromethane-methanol) to obtain **9b** (322 mg, 83.20%) as a white solid; m.p. 151.1–152.9 °C; ^1^H-NMR (600 MHz, C_5_D_5_N) *δ* 11.77 (s, 1H), 9.95 (s, 1H), 9.42 (s, 1H), 8.93 (s, 2H), 8.23 (d, *J* = 7.8 Hz, 2H), 8.14 (d, *J* = 7.8 Hz, 2H), 6.52 (s, 1H), 4.71 (q, *J* = 18.4, 9.2 Hz, 1H), 4.49 (d, *J* = 10.9 Hz, 1H), 4.32 (s, 1H), 4.31 (s, 1H), 4.11 (t, *J* = 9.1 Hz, 1H), 4.04 (s, 1H), 2.05 (s, 3H); ^13^C-NMR (150 MHz, C_5_D_5_N) *δ* 183.79, 173.79, 143.86, 140.92, 127.48, 123.49, 85.05, 80.50, 76.55, 72.26, 62.63, 56.54, 23.33; HRMS (ESI): Calcd. for [M + Na]^+^ C_15_H_22_N_4_Na_1_O_7_S_2_: 457.0828, Found 457.0822.

### 3.3. Carbonic Anhydrase Inhibition Assay

Carbonic anhydrases are able to catalyze the conversion 4-nitrophenyl acetate (4-NPA) to 4-nitrophenol. According to the reported method [36], the rate of this reaction is monitored spectrophotometrically, at 405 nm, with a Perkin Elmer Envision 2104 plate reader. One × Assay buffer is composed of 15 mmol/L 4-(2-hydroxyethyl)-1-piperazineethanesulfonic acid (HEPES) (pH = 7.4), 0.01% tetraethylene glycol monododecyl ether (BRIJ) and 100 mmol/L NaCl. Recombinant human carbonic anhydrase IX was commercially available (Sino Biological Inc, Beijing, China) and prepared in 1 × assay buffer with a concentration of 11.1 ng/μL. Then, 18 μL of enzyme solution was transferred into 384-well assay plates in triplicate. Stock solutions of the inhibitor (10 mmol/L) were prepared using DMSO as solvent, and then diluted 1:3 with DMSO. Ten different inhibitor concentrations were used: 600 μmol/L, 200 μmol/L, 66.67 μmol/L, 22.22 μmol/L, 7.41 μmol/L, 2.47 μmol/L, 0.82 μmol/L, 0.27 μmol/L, 0.091 μmol/L, 0.03 μmol/L, and 0 μmol/L. A volume of 2 μL of each inhibitor was added into the assay solution. All compounds were allowed to incubate with the enzyme for 15 min at 25 °C to form the Enzyme-Inhibitor (E-I) complex. After that, substrate 4-NPA (1 mmol/L, 20 μL, Sigma-Aldrich, St. Louis, MI, USA) was added into the E-I complex solution and incubated for 90 min at 25 °C. The absorbance of each compound was measured with Envision 2104 plate reader. The normalized data were then fit to a sigmoidal dose-response curve using GraphPad Prism 5.0 software, La Jolla, CA, USA to obtain the IC_50_. Final enzyme concentration was 5 ng/μL. The final concentrations of the inhibitors were 30 μmol/L, 10 μmol/L, 3.3333 μmol/L, 1.1111 μmol/L, 0.3704 μmol/L, 0.1235 μmol/L, 0.0412 μmol/L, 0.0137 μmol/L, 0.0046 μmol/L, 0.0015 μmol/L, and 0 μmol/L. The substrate and DMSO final concentrations were 0.5 mmol/L and 5%, respectively 4.

## 4. Conclusions

In conclusion, we report a novel series of *N*-substituted-*β*-d-glucosamines derivatives incorporating benzenesulfonamides by utilizing a fragment-based drug design. The rationale behind this design is demonstrated by the molecular docking study. Each compound was evaluated for its ability to inhibit membrane-associated CA IX in vitro, and all compounds exhibited activities against the enzyme to varying degrees. Several substituents that contained sulfonyl moieties on the 2-amino group of their glucosamines were better inhibitors of CA IX than is AZA. It is a challenge to have compounds with potency and specificity for CA IX based on the conserved three-dimensional architecture of CA isoforms, however, these potent glycoconjugates with membrane impermeability may provide the opportunities to target extracellular isozyme CA IX. This finding is greatly encouraging for us to exploit highly potent, selective, and drug-like lead compound CA IX inhibitors.

## Figures and Tables

**Table 1:**
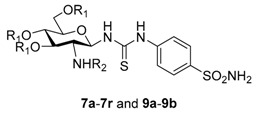
Molecular docking binding scores, calculated Log P (cLog P) data, topological polar surface area (TPSA), and inhibition of human carbonic anhydrase (hCA) IX for target compounds **7a**–**7r** and **9a**–**9b**.

Compound	R_1_	R_2_	Docking Scores (Kcal/mol)		cLog P	TPSA	hCA IX (IC_50_, nM) ^a,b^
			Autodock	X-Score			
**AZA ^c^**			−6.34	−6.81	−0.9809	113.77	21.52 ± 2.36
**7a**	H	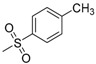	−7.46	−8.56	0.1321	205.55	21.75 ± 3.18
**7b**	H	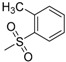	−7.67	−9.03	0.1321	205.55	21.90 ± 0.37
**7c**	H	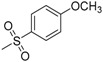	−7.38	−8.39	−0.0893	214.48	23.78 ± 0.51
**7d**	H	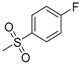	−7.10	−8.33	0.0785	205.55	25.57 ± 2.15
**7e**	H	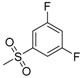	−7.05	−8.08	0.3273	205.55	11.47 ± 0.93
**7f**	H	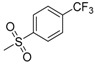	−7.10	−8.66	1.0453	205.55	10.01 ± 0.23
**7g**	H	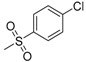	−7.17	−8.75	0.6485	205.55	26.85 ± 0.28
**7h**	H	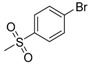	−7.53	−8.81	0.7985	205.55	21.34 ± 1.29
**7i**	H	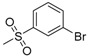	−7.52	−8.72	0.7985	205.55	12.56 ± 0.96
**7j**	H	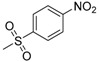	−8.63	−8.51	−3.6589	248.37	25.42 ± 3.80
**7k**	H	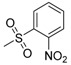	−8.51	−8.23	−3.6589	248.37	15.63 ± 0.44
**7l**	H	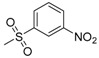	−8.17	−7.52	−0.2319	248.37	18.95 ± 0.33
**7m**	H	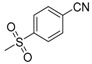	−7.74	−7.46	0.8071	228.49	21.86 ± 0.49
**7n**	H	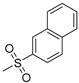	−8.15	−8.18	−1.0982	205.55	24.78 ± 0.05
**7o**	H	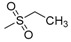	−6.69	−8.14	−0.5692	205.55	66.88 ± 3.26
**7p**	H	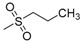	−6.58	−7.94	−1.1367	205.55	388.80 ± 27.70
**7q**	H	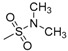	−7.39	−8.18	−0.4524	208.90	72.09 ± 6.15
**7r**	H	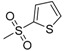	−7.43	−8.33	−3.6589	205.55	65.80 ± 0.08
**9a**	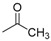	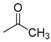	−7.61	−8.05	−0.6134	188.25	1292.00 ± 141.35
**9b**	H	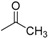	−6.16	−8.68	−1.6898	204.50	175.80 ± 9.45

^a^ IC_50_ values were determined by the 4-nitrophenyl acetate (4-NPA) esterase assay. ^b^ The data are expressed in the form of mean ± standard error. ^c^ Acetazolamide (AZA) has been included as a positive control.
